# What happens in plants stays in plants: Arabidopsis prime Ac/N-recognin candidates do not function as such

**DOI:** 10.1093/plphys/kiad450

**Published:** 2023-08-09

**Authors:** Manuel González-Fuente

**Affiliations:** Assistant Features Editor, Plant Physiology, American Society of Plant Biologists; Faculty of Biology & Biotechnology, Ruhr-University Bochum, 44780 Bochum, Germany

Even the smallest genomes generate highly sophisticated organisms. This is possible because a single genome encodes millions of different proteomes. This versatility is achieved through a complex spatio-temporal regulation at the transcriptional, posttranscriptional, translational, and posttranslational levels. It is the latter, the posttranslational modifications of proteins, that contributes the most to the proteome complexity (Spoel 2018).

Posttranslational modifications of proteins alter their chemical properties, which ultimately determines their structure, stability, multimerization status, localization, and function. Although there are hundreds of different posttranslational modifications, acetylation of N-termini (NTA) is one of the most prevalent, occurring in more than 80% of animal and plant proteins (Bienvenut et al. 2012). As the name indicates, NTA consists of the addition of an acetyl group to the first amino acid of a protein.

In yeast and humans, one of the possible outcomes of the NTA of a protein is its proteasomal degradation via the so-called acetylation-dependent (Ac/) N-degron pathway (Hwang et al. 2010; Langin et al. 2023). This pathway relies on the recognition of N-terminally acetylated proteins by specific E3 ligases named Ac/N-recognins (Fig. 1). The best characterized examples of such recognins are the yeast *Sc*DOA10 and its human ortholog *Hs*MARCH6 (Hwang et al. 2010; Nguyen et al. 2019). Although there have been hints that the Ac/N-degron pathway might also exist in plants (Xu et al. 2015), until now not a single plant Ac/N-recognin has been identified.

**Figure 1.**
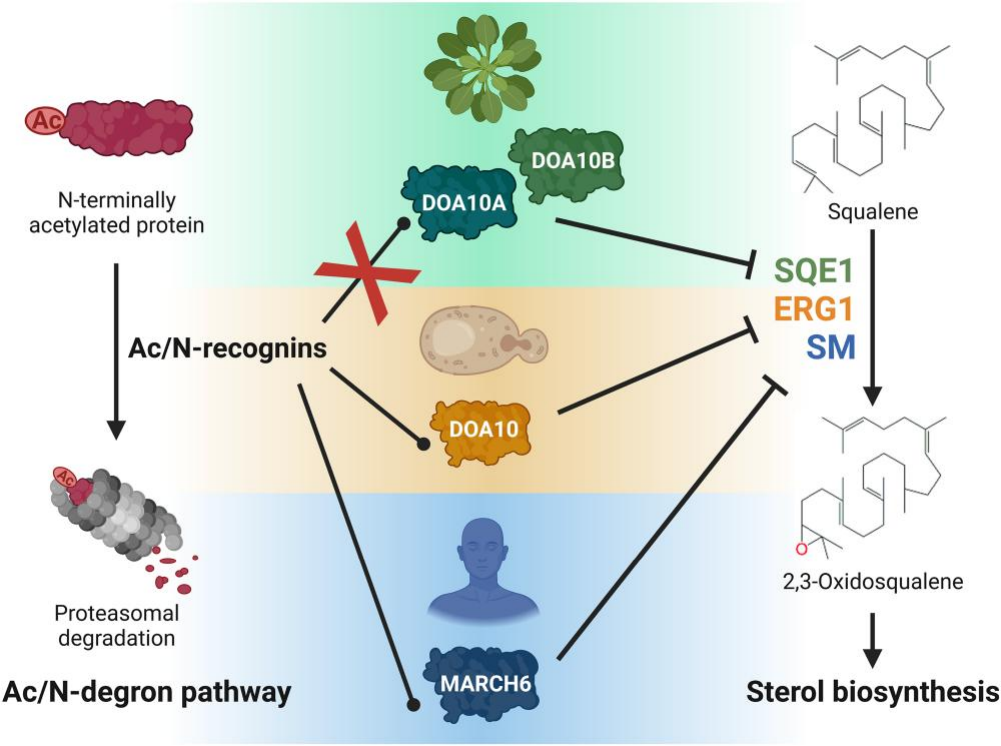
Arabidopsis DOA10s do not function as Ac/N-recognins in plants but still control the turnover of a conserved key enzyme in sterol biosynthesis. Arabidopsis *At*DOA10A and *At*DOA10b, unlike their yeast and human counterparts (*Sc*DOA10 and *Hs*MARCH6, respectively), do not function as recognins for the Ac/N-degron pathway (left). Nevertheless, both paralogs stills regulate the turnover of SQE1, a rate-limiting enzyme of the sterol biosynthetic pathway whose ortholog in yeast and human (ERG1 and SM, respectively) are similarly controlled by DOA10-like proteins (right). Created with BioRender.com.

In this issue of *Plant Physiology*, Etherington et al. (2023) explore whether *At*DOA10A and *At*DOA10B, the Arabidopsis orthologs of *Sc*DOA10/*Hs*MARCH6, act as plant Ac/N-recognins. As an initial approach to determine their possible role as Ac/N-recognins, the authors cross-complemented a yeast *Scdoa10* mutant strain with either *AtDOA10A* or *AtDOA10B*. *AtDOA10A*, but not *AtDOA10B*, complemented the *Scdoa10* mutation. This correlates with *AtDOA10A* having a higher degree of homology to *ScDOA10* and greater conservation among land plants. Moreover, similar to their yeast and animal counterparts, both *At*DOA10A and *At*DOA10B also localized to the endoplasmic reticulum. Altogether, these similarities pointed toward a possible role of *At*DOA10s, or at least *At*DOA10A, as Ac/N-recognins.

If indeed *At*DOA10s acted as general Ac/N-recognins, the double *Atdoa10a/b* mutant would accumulate more N-acetylated proteins. However, the proportion of acetylated N-termini identified in the double mutant was comparable with the wild type. Moreover, when analyzing the percentage of acetylation of N termini depending on their amino acid composition, position in the peptide, or subcellular localization of the protein, no significant differences were observed between the double mutant and the wild type. These data suggest that *At*DOA10s do not have a major role as *general* recognins of the Ac/N-degron pathway (Fig. 1). Nevertheless, the data do not exclude the possibility that *At*DOA10s could still target *specific* N-acetylated proteins for degradation.

To explore this possible role of *At*DOA10s as specific Ac/N-recognins of certain proteins, the authors used a targeted approach, looking for known common substrates of *Sc*DOA10 and *Hs*MARCH6. Both Ac/N-recognins target the respective yeast and human ortholog of the Arabidopsis SQUALENE EPOXIDASE 1 (*At*SQE1), a rate-limiting enzyme in the sterol biosynthesis pathway, which is also located at the endoplasmic reticulum (Foresti et al. 2013). Interestingly, *At*DOA10A was previously linked to sterol biosynthesis (Doblas et al. 2013), thus making *At*SQE1 an ideal candidate target of the Ac/N-degron pathway mediated by *At*DOA10s.

Indeed, *At*SQE1 protein levels accumulated in the *Scdoa10* mutant when heterologously expressed in yeast (Etherington et al. 2023). Moreover, this accumulation was compromised when *At*DOA10A, but not *At*DOA10B, was coexpressed, indicating that heterologous *At*DOA10A controls the turnover of *AtSQE1* in yeast. Counterintuitively, this *At*DOA10A-mediated degradation of *At*SQE1 in yeast is independent of its N-acetylation status, as *At*SQE1 variants unable to be N-terminally acetylated are still similarly degraded. This result shows that, although *At*DOA10A indeed controls the turnover of *At*SQE1 protein levels in yeast, it is not by directly targeting its acetylated N terminus but rather through a yet-unknown mechanism.

As the previous studies were conducted in a heterologous system, the authors next analyzed the stability of *At*SQE1 in planta using transgenic Arabidopsis lines. They observed that *At*SQE1 protein levels were stabilized only in the double *Atdoa10a/b* mutant, demonstrating the redundant role of both paralogs in controlling the turnover of *At*SQE1. This functional redundancy of *At*DOA10A and *At*DOA10B in Arabidopsis marks a difference with the heterologous yeast system in which only *At*DOA10A controlled the turnover of *At*SQE1. Nevertheless, similar to yeast, in Arabidopsis *At*SQE1 protein stability was still not determined by its N-acetylation status, as the *At*SQE1 variant unable to be N-terminally acetylated was also equally degraded in the plant. Additionally, the authors observed that the *Atdoa10/b* double mutant leaves were bent downward, a phenotype typically associated with altered sterol biosynthesis (Carland et al. 2010), linking *At*SQE1 overaccumulation with alterations in phytosterol homeostasis. Altogether, these results show that *At*DOA10s regulate sterol biosynthesis pathways in Arabidopsis by modulating the degradation of *At*SQE1 in a NTA-independent manner (Fig. 1).

In summary, in this study Etherington et al. (2023) showed that similarly to *Sc*DOA10 in yeast and *Hs*MARCH6 in humans (Foresti et al. 2013), *At*DOA10s also regulate sterol homeostasis in plants by controlling the turnover of the same key enzyme in the sterol biosynthesis pathway. Nevertheless, despite this similarity and the conservation of DOA10 proteins in eukaryotes, the molecular mechanisms behind their regulatory role could differ as *Sc*DOA10 and *Hs*MARCH6 are well-characterized Ac/N-recognins (Hwang et al. 2010; Nguyen et al. 2019), whereas *At*DOA10s do not seem to be (Fig. 1).

The results presented in Etherington et al. (2023) reveal both substantial differences and yet important similarities in DOA10s across kingdoms. Even though their homology and subcellular localization made *At*DOA10s ideal candidates to function as Ac/N-recognins in plants, this study demonstrates that this is not the case. The results presented in this study raise several questions, such as whether there are other plant proteins that can function as Ac/N-recognins or what is the importance and extent of the Ac/N-degron pathway in plants, whose mere existence has been debated for long (Xu et al. 2015; Etherington et al. 2023). Moreover, even though it is NTA independent, *At*DOA10s still control the turnover of *At*SQE1 in plants through a mechanism that remains to be determined. This work also reminds us as plant scientists that we need to be cautious and avoid automatically assuming that the same molecular mechanisms described in other eukaryotes necessarily occur in plants.

## Dive Curated Terms

The following phenotypic, genotypic, and functional terms are of significance to the work described in this paper:

SQUALENE CHEBI: CHEBI:15440

AtSQE1 Gramene: At1g58440
AtSQE1 Araport: At1g58440

AtDOA10A Gramene: At4g34100
AtDOA10A Araport: At4g34100

AtDOA10B Gramene: At4g32670
AtDOA10B Araport: At4g32670
